# Epidemiological and genetic characteristics of vancomycin-resistant *Enterococcus faecium* isolates in a University Children's Hospital in Germany: 2019 to 2020

**DOI:** 10.1186/s13756-022-01081-3

**Published:** 2022-03-12

**Authors:** Ilona Trautmannsberger, Laura Kolberg, Melanie Meyer-Buehn, Johannes Huebner, Guido Werner, Robert Weber, Valerie Heselich, Sebastian Schroepf, Hans-Georg Muench, Ulrich von Both

**Affiliations:** 1 Division of Paediatric Infectious Diseases, Dr. Von Hauner Children’s Hospital, University Hospital, Ludwig-Maximilians-University (LMU) Munich, Lindwurmstr. 4, 80337 Munich, Germany; 2 Institute for Medical Information Processing, Biometry, and Epidemiology – IBE, LMU Munich, Munich, Germany; 3 Pettenkofer School of Public Health, Munich, Germany; 4 German Center for Infection Research (DZIF), Partner Site Munich, Munich, Germany; 5 Robert Koch Institute, Wernigerode Branch, Wernigerode, Germany; 6 Helmholtz Centre for Infection Research, Braunschweig, Germany; 7 Division of Neonatology, Dr. Von Hauner Children’s Hospital, University Hospital, LMU Munich, Munich, Germany

**Keywords:** Vancomycin-resistant *Enterococcus faecium*, Paediatrics, Epidemiology, Outbreak investigation, Nosocomial cluster, Germany

## Abstract

**Background:**

Vancomycin-resistant *Enterococcus faecium* (VREfm) strains are one of the most important pathogens causing nosocomial infections in Germany. Due to limited treatment options and an increased risk for acquisition in immunocompromised children, surveillance to monitor occurrence of VREfm in paediatric clinical facilities is of critical importance. Following an unusual accumulation of VREfm positive patients between April 2019 and August 2020 at Dr. von Hauner Children’s Hospital in Munich, Germany, our study aimed to identify dynamics and routes of transmission, and analyse the affected population in view of previously described host risk factors for VREfm colonisation or infection.

**Methods:**

The hospital database was used to collect epidemiological and clinical data of VREfm cases. Descriptive statistical analyses were conducted to outline patient characteristics and depict possible differences between VREfm-colonised and -infected children. An outbreak investigation determining genetic relatedness among VREfm isolates was performed by core genome multilocus sequence typing (cgMLST). To examine potential transmission pathways, results of genome analysis were compared with epidemiological and clinical data of VREfm positive patients.

**Results:**

VREfm acquisition was documented in a total of 33 children (< 18 years). Seven VREfm-colonised patients (21.2%), especially those with a haemato-oncological disease (4/7; *p* = 0.011), showed signs of clinical infection. cgMLST analysis revealed seven distinct clusters, demonstrating a possible connection within each clonal lineage. Additional eight singletons were identified. Comparison with epidemiological and clinical data provided strong evidence for a link between several VREfm positive patients within the hospital.

**Conclusions:**

A nosocomial spread—at least in part—was the most likely reason for the unusual accumulation of VREfm cases. The study highlights that there is a constant need to increase efforts in hygiene measures, infection control and antibiotic stewardship to combat VREfm transmission events within German paediatric hospitals. Continuous monitoring of adherence to respective policies might reduce the occurrence of clustered cases and prevent future outbreaks.

## Background

Enterococci are Gram-positive, catalase-negative, facultative anaerobic bacteria that commonly inhabit the gastrointestinal tract of humans and animals. Of all *Enterococcus* species known to date, *Enterococcus faecalis* and *E. faecium* are the most common commensal organisms in humans [1]. Both are characterised by great tenacity to hostile environmental conditions, including high NaCl concentration, bile salts, pH (4.5–10.0) and extreme temperature (5–65 °C), enabling them to persist, grow and spread under a range of stresses [1, 2]. In addition to their role as an essential part of the microflora, *E. faecium* and *E. faecalis* are of great medical significance. They are important nosocomial pathogens causing a variety of infections, such as urinary tract and surgical site infections, peritonitis, bacteraemia and in severe cases bloodstream infections and endocarditis [3–6]. A major challenge is the occurrence of intrinsic and acquired antibiotic resistance, which significantly reduces possibilities for therapy. In particular, the glycopeptide resistance genotypes *vanA* and *vanB* in vancomycin-resistant *E. faecium* (VREfm) isolates cause fundamental therapeutic problems [4, 6–8].

Considering the increased risk for persistence and transmission in hospitals, VREfm infections and their treatment play an important role in clinical practice [1, 2, 9–11]. Since the beginning of the twenty-first century, an increased spread of *vanA*- and *vanB*-positive *E. faecium* strains has been detected in German hospitals [5, 12, 13]. This resulted in major outbreaks of VREfm infections and colonisations, which led to a continuous expansion of resistance rates in subsequent years [5, 12–14]. According to a recent analysis of data on *E. faecium* isolates from the Antibiotic Resistance Surveillance (ARS) of the Robert Koch Institute (RKI), the proportion of existing vancomycin resistance in German hospitals increased significantly from 11.2% in 2014 to 26.1% in 2017 [15]. Due to proven evidence on prolonged hospital stay, higher costs and excess mortality amongst VREfm-colonised and -infected patients, the World Health Organization (WHO) assigned VREfm as a high priority pathogen on its global priority list of antibiotic-resistant bacteria [16–18].

Despite the fact that paediatric facilities are currently not considered classic risk areas for VREfm occurrence, hospitalised children and neonates especially those with severe comorbidities are highly susceptible for VREfm acquisition, colonisation and subsequent infection after contact with these bacteria [19–25]. Possessing immunological naivety and requiring intensive care, they present a fundamentally vulnerable patient group, for whom infections remain an important cause of death [22, 23, 26]. Therefore, it is of great interest to investigate frequent occurrence of VREfm in neonatal, interdisciplinary paediatric and paediatric surgical facilities, examine the affected population and identify spread dynamics. Potential VREfm clusters can thus be detected and current measures for prevention and control of healthcare-associated infections can be reviewed, adapted and improved.

Between April 2019 and August 2020, an unusual accumulation of VREfm cases was observed at Dr. von Hauner Children’s Hospital in Munich, Germany. The aim of the study was to identify or exclude a clonal spread, determine possible nosocomial transmission routes, analyse the affected population in view of previously described host risk factors for VREfm colonisation or infection, give suggestions to improve prevention measures and thereby reduce the rate of future VREfm-colonised and -infected patients at Dr. von Hauner Children’s Hospital.

## Methods

### Study design and study population

This study was designed as a monocentric, descriptive retrospective analysis investigating data of children aged < 18 years with acquired VREfm isolates between April 2019 and August 2020. The analysis focused on both colonised and infected patients at Dr. von Hauner Children’s Hospital, a 180-bed paediatric tertiary teaching hospital in Munich, Germany. As a part of the Ludwig-Maximilians-University (LMU) Klinikum, it combines general paediatrics and paediatric surgery, provides outpatient care and treats about 7500 inpatient cases every year [27]. Following local proximity and a high number of patient referrals, affected newborns on the neonatal intensive care unit (NICU) in the Polyclinic for Gynecology and Obstetrics (LMU Klinikum Campus Inner City) were included as well. During the study period, the bacteriological laboratory of Dr. von Hauner Children’s Hospital isolated VREfm from 33 patients in total. Cases of VREfm (colonisation or infection) were identified either by a microbiological analysis of rectal swabs examined due to screening for multidrug-resistant pathogens or any other clinical specimen tested for presumed bacterial infection. Routine screening using rectal swabs was performed on NICU and PICU (paediatric intensive care unit) on a weekly basis and on any patient newly admitted to these wards (starting August 2020). Rectal swabs were directly applied to VRE selection agar plates (VRE Select, reference number 63751, Bio-Rad, 85622 Feldkirchen, Germany).

### VREfm isolates and cgMLST

VREfm isolates detected at the Dr. von Hauner Children’s Hospital were sent to the German National Reference Centre for Staphylococci and Enterococci at the Robert Koch Institute for further analysis. Antibiotic susceptibility testing was performed by broth microdilution and subsequent determination of the minimum inhibitory concentrations according to the European Committee on Antimicrobial Susceptibility Testing (EUCAST) guidelines and breakpoints (v10) [28]. Species identification and detection of resistance genes (*vanA, vanB*) were conducted using standard polymerase chain reaction (PCR) assays. An outbreak investigation determining possible clonal relatedness among the isolates was initiated by whole-genome sequencing (WGS) and typing. For this purpose, DNA derived from pure bacterial culture was isolated using the DNeasy Blood & Tissue Kit (Qiagen, Hilden, Germany). Sequencing libraries were generated with the Nextera XT DNA Library Preparation Kit (Illumina, San Diego, CA, United States) and paired-end sequencing was performed using a NextSeq instrument with a read length of 150 bp (Illumina, San Diego, CA, United States). The quality of the raw sequence data was checked using FastQC v0.11.5 [29]. Additionally, Kraken v0.10.6 was used to verify taxonomic read classification [30]. Subsequently, SPAdes v3.12.0 was used in the assembly mode ‘careful’ with default parameters for de novo assembly of high-quality sequencing reads [31]. Multilocus sequence typing (MLST) and core genome MLST (cgMLST) were performed using contigs and Ridom SeqSphere + v6.0.0 (Ridom; Münster, Germany) and established typing schemes [32, 33]. Sequence types (ST) and complex types (CT) were derived from MLST and cgMLST, respectively. Based on cgMLST (including 1423 core genes), a Minimum Spanning Tree (MST) was inferred by ignoring pairwise missing values. VREfm isolates, assigned to the same *van*-genotype and differing in less than 15 core genes were considered as (closely) related [34].

### Data collection and variables

Epidemiological and clinical data of the study population were extracted from electronic and paper-based medical records provided by the hospital database. Variables collected were age at initial detection of VREfm, sex, hospital wards patients were present during the study period and information about whether the patient had a presumed clinical infection or was colonised. Predisposition to known risk factors was identified by literature search and included in the analysis. In addition to multiple patient-related factors such as preterm birth (including young gestational age and low gestational weight), underlying immunosuppressive comorbidity (e.g. malignancy), performed surgical procedures, use of invasive devices (e.g. catheters and feeding or breathing tubes) and invasive treatments (ventilation, chemotherapy), the exposition to antibiotics was recorded [19, 20, 23, 24, 35–38]. Antibiotics prescribed within six months before initial VREfm detection were analysed and categorised into antibiotic classes and *Access*, *Watch*, or *Reserve* groups according to the WHO AWaRe classification of antibiotics [39]. In case of a suspected infection, the respective antibiotic used for treatment was included in the analysis.

### Statistical analysis

Statistical analysis was performed using R version 4.0.5 [40]. Distribution of categorical variables in the study population was described in absolute numbers and percentages, continuous variables were illustrated with measures (median, range). Patient characteristics were further analysed regarding suspected VREfm infection (VREfm-I) and VREfm colonisation (VREfm-C). Parameters were compared with Fisher’s exact test for categorical variables and Mann–Whitney U test for continuous variables, as all quantitative data were not normally distributed. The significance level was set at 5%. Missing values were excluded for analysis. A timeline was generated to combine results from cgMLST with epidemiological and clinical data of the study population and thereby investigate potential transmission pathways within the hospital.

## Results

**Fig. 1:**
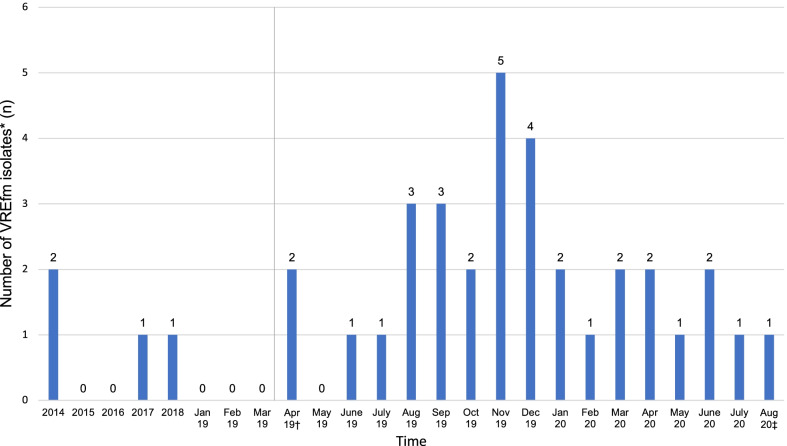
Time trend of detected vancomycin-resistant *E. faecium* isolates from 2014 to August 2020. ^†^beginning of the study period at Dr. von Hauner Children’s Hospital, ^‡^end of the study period at Dr. von Hauner Children’s Hospital

Between April 2019 and August 2020, a total of 693 children were screened for VREfm (154 (22.2%) children in non-ICU settings and 539 (77.8%) while being treated on intensive care units (NICU, PICU)). 33/693 patients were found to be colonised, accounting for a prevalence of 4.8%. Compared to previous years, the number of detected VREfm isolates showed a notable increase during the study period (see Fig. 1). Detailed baseline characteristics of children found to be VREfm positive are outlined in Table 1. Cases predominantly originated from infants with a median age of 6 months (range 0–16 years) and a male/female ratio of 1.54. At the time of VREfm detection, 26 children were treated on neonatal/paediatric intensive care units, four were identified on surgical wards, one on the bone marrow transplantation unit and two were patients cared for on general wards. Antibiotics prescribed within six months prior to detection of VREfm are shown in Table 2.

**Table 1:** Demographic and clinical data of the study population (patients < 18 years)

	Missing n (%)	All patients (n = 33)	VREfm-colonised patients (n = 26)	VREfm-colonised patients with suspected infection (n = 7)	*p* value*
**Sex, n (%)**	0 (0.0)				
Male		20 (60.6)	17 (65.4)	3 (42.9)	0.393
Female		13 (39.4)	9 (34.6)	4 (57.1)	
**Age (in months)**	0 (0.0)				
Median (range)		6 (0–198)	5.5 (0–198)	13 (1–184)	0.143
**Prematurity, n (%)**	4 (12.1)				
Preterm born		16 (48.5)	14 (53.8)	2 (28.6)	0.632
Mature born		13 (39.4)	10 (38.5)	3 (42.9)	
**Gestational age (in completed weeks of gestation)**	4 (12.1)				
Median (range)		34 (23–41)	33.5 (23–41)	34 (24–40)	0.885
**Gestational weight (in grams)**	8 (24.2)				
Median (range)		2150 (465–4430)	2150 (465–4430)	1665 (730–2600)	0.96
**Twin, n (%)**	0 (0.0)				
Yes		4 (12.1)	4 (15.4)	0 (0.0)	0.555
**Invasive devices**^†^**, n (%)**	0 (0.0)				
NG/NJ tube		16 (48.5)	13 (50.0)	3 (42.9)	1.000
PEG/PEJ tube		7 (21.2)	6 (23.1)	1 (14.3)	1.000
Peripheral venous catheter		21 (63.6)	15 (57.7)	6 (85.7)	0.223
Central venous catheter		17 (51.5)	13 (50.0)	4 (57.1)	1.000
Arterial line		9 (27.3)	6 (23.1)	3 (42.9)	0.358
Port		1 (3.0)	1 (3.8)	0 (0.0)	1.000
Hickman catheter		5 (15.2)	2 (7.7)	3 (42.9)	0.052
Urinary catheter		10 (30.3)	6 (23.1)	4 (57.1)	0.161
Ventricular drain		3 (9.1)	3 (11.5)	0 (0.0)	1.000
Tracheostomy tube		2 (6.1)	2 (7.7)	0 (0.0)	1.000
Chest drain		2 (6.1)	2 (7.7)	0 (0.0)	1.000
Artificial stoma		3 (9.1)	0 (0.0)	3 (42.9)	**0.006**
**Surgical procedures**^†^**, n (%)**	0 (0.0)	18 (54.5)	11 (42.3)	7 (100.0)	**0.009**
Endoscopic procedure		8 (24.2)	7 (26.9)	1 (14.3)	0.652
Cardiothoracic surgery		4 (12.1)	3 (11.5)	1 (14.3)	1.000
Intrabdominal surgery		5 (15.2)	0 (0.0)	5 (71.4)	**< 0.0001**
Brain surgery		2 (6.1)	2 (7.7)	0 (0.0)	1.000
Biopsy		2 (6.1)	1 (3.8)	1 (14.3)	0.385
Bone marrow aspiration		1 (3.0)	0 (0.0)	1 (14.3)	0.212
Tumour resection		3 (9.1)	0 (0.0)	3 (42.9)	**0.006**
Other surgical procedures^‡^		2 (6.1)	1 (3.8)	1 (14.3)	0.385
**Underlying diseases, n (%)**	0 (0.0)				
Haemato-oncological diseases		6 (18.2)	2 (7.7)	4 (57.1)	**0.011**
Cardiovascular diseases		8 (24.2)	6 (23.1)	2 (28.6)	1.000
Diseases of the respiratory system		13 (39.4)	13 (50.0)	0 (0.0)	**0.027**
Endocrine diseases		3 (9.1)	3 (11.5)	0 (0.0)	1.000
Gastrointestinal diseases		15 (45.5)	11 (42.3)	4 (57.1)	0.674
Genitourinary diseases		3 (9.1)	1 (3.8)	2 (28.6)	0.107
Neurological diseases		9 (27.3)	8 (30.8)	1 (14.3)	0.642
Malformation syndromes affecting multiple systems		3 (9.1)	2 (7.7)	1 (14.3)	0.524
Chromosomal abnormalities		3 (9.1)	3 (11.5)	0 (0.0)	1.000
**Ventilation**^†^**, n (%)**	0 (0.0)				
Invasive ventilation		14 (42.4)	10 (38.5)	4 (57.1)	0.422
Non-invasive ventilation		17 (51.5)	13 (50.0)	4 (57.1)	1.000
**Chemotherapy**^⁓^**, n (%)**	0 (0.0)				
Yes		4 (12.1)	1 (3.8)	3 (42.9)	**0.023**
**Reanimation**^⁓^**, n (%)**	0 (0.0)				
Yes		5 (15.2)	3 (11.5)	2 (28.6)	0.282
**Overall hospitalisation before initial detection of VREfm**^**°**^	0 (0.0)				
Length of stay (in days), median (range)		38 (0–276)	38 (0–276)	28 (0–106)	0.659
Number of hospital admissions, median (range)		1 (1–34)	1 (1–34)	1 (1–15)	0.484
**Glycopeptide resistance genotype, n (%)**	0 (0.0)				
vanA		15 (45.5)	14 (53.8)	1 (14.3)	0.095
vanB		18 (54.5)	12 (46.2)	6 (85.7)	

Signficant values (marked in bold) were defined as *p* < 0.05

VREfm, Vancomycin-resistant *Enterococcus faecium*; NG/NJ tube, nasogastric/nasojejunal tube; PEG/PEJ tube, percutaneous endoscopic gastrostomy/jejunostomy tube

^*^Fisher’s exact test or Mann–Whitney U test (p $\le$ 0.05 was considered significant)

^†^Within four weeks prior to detection of VREfm

^‡^Including one surgery of the anus, rectum and colon and one ovarian surgery for fertility preservation

^⁓^Within six months prior to detection of VREfm

°Including hospitalisation at Dr. von Hauner Children’s Hospital, NICU Clinic and Polyclinic for Gynecology and Obstetrics and LMU Klinikum Großhadern

**Table 2:** Antibiotic use within six months prior to detection of VREfm in the study population

	All patients (n = 33)	VREfm-colonised patients (n = 26)	VREfm-colonised patients with suspected infection (n = 7)	*p* value*
**Antibiotics total**, median (range)	5 (0–14)	5 (0–14)	7 (3–14)	0.150
**Antibiotic classes, n (%)**				
Aminoglycosides	5 (15.2)	5 (19.2)	0 (0.0)	0.559
Beta-lactam/beta-lactamase inhibitor	22 (66.7)	16 (61.5)	6 (85.7)	0.378
Carbapenems	16 (48.5)	10 (38.5)	6 (85.7)	**0.039**
First-generation cephalosporins	3 (9.1)	3 (11.5)	0 (0.0)	1.000
Fluoroquinolones	1 (3.0)	0 (0.0)	1 (14.3)	0.212
Glycopeptides	11 (33.3)	9 (34.6)	2 (28.6)	1.000
Imidazoles	4 (12.1)	3 (11.5)	1 (14.3)	1.000
Macrolides	6 (18.2)	6 (23.1)	0 (0.0)	0.301
Penicillins	12 (36.4)	11 (42.3)	1 (14.3)	0.223
Phosphonics	1 (3.0)	0 (0.0)	1 (14.3)	0.212
Second-generation cephalosporins	8 (24.2)	7 (26.9)	1 (14.3)	0.652
Third-generation cephalosporins	14 (42.4)	13 (50.0)	1 (14.3)	0.195
Trimethoprim/sulfonamide combinations	9 (27.3)	5 (19.2)	4 (57.1)	0.068
Unknown antibiotic class	2 (6.1)	2 (7.7)	0 (0.0)	1.000
**Antibiotic groups AWaRe classification**^†^				
Access Antibiotics, median (range)	1 (0–6)	1 (0–6)	1 (0–3)	0.629
Watch Antibiotics, median (range)	4 (0–13)	3 (0–13)	4 (2–12)	0.120

Signficant values (marked in bold) were defined as *p* < 0.05

VREfm, Vancomycin-resistant *Enterococcus faecium*

^*^Fisher’s exact test or Mann–Whitney U test (*p*  < 0.05 was considered significant)

^†^excluding two unknown antibiotics

A clinical VREfm infection was presumed in seven patients (21.2%), all of whom were previously colonised with the respective VREfm strain. Central-line associated infection was detected in three patients. Two patients suffered a surgical wound infection, one patient was diagnosed with a urinary tract infection and another one showed positive blood cultures, suggesting an invasive systemic infection. Demographic and clinical findings from patients with suspected VREfm-I and VREfm-C are presented in Table 1. Our data demonstrate that children who developed signs of a bacterial infection were significantly more likely to have had a temporary artificial stoma (*p* = 0.006), to have undergone a recent surgical procedure (*p* = 0.009) or to have received carbapenems (*p* = 0.039) or chemotherapy (*p* = 0.023) before initial VREfm detection. Similarly, the presence of an underlying haemato-oncological diagnosis (*p* = 0.011) was significantly more likely in VREfm-infected compared to VREfm-colonised children. An underlying respiratory disease (*p* = 0.027) was significantly less likely in patients with a presumed VREfm-I than in those without clinical symptoms.

All suspected VREfm infections were treated with *Reserve* antibiotics. Six patients received linezolid, one patient received daptomycin and another child was treated with a linezolid/daptomycin combination.

**Fig. 2:**
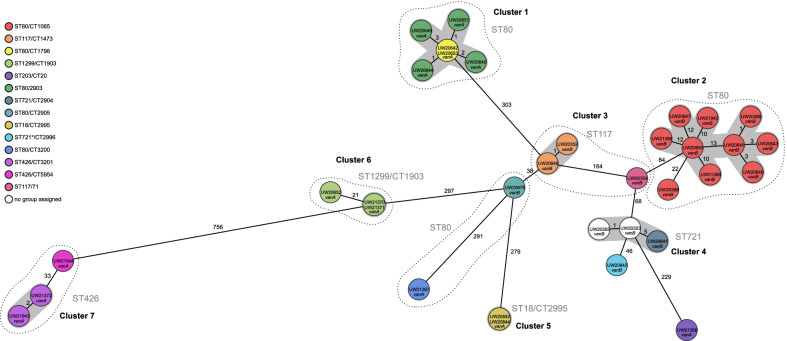
Minimum spanning tree of 34 vancomycin-resistant *E. faecium* isolates from 33 children and one mother. The number of varying alleles is shown next to the black lines. Colouring was based on cgMLST (analysis of 1423 genes of the nuclear genome). Grey areas connect isolates that belong to a cluster based on the definitions of SeqSphere + (maximum allele difference = 15 alleles). Dashed lines include isolates that belong to one sequence type (ST). ST = sequence type (multilocus sequence typing (MLST), analysis of 7 housekeeping genes), CT = complex type (core genome MLST (cgMLST)). SeqSphere option: pairwise ignoring missing values. Task templates: *E. faecium* cgMLST v1.1; *E. faecium* MLST v1.0. *adk: Novel allele, ST may indicate nearest ST. MLST-Finder: http://www.genomicepidemiolog

**Fig. 3:**
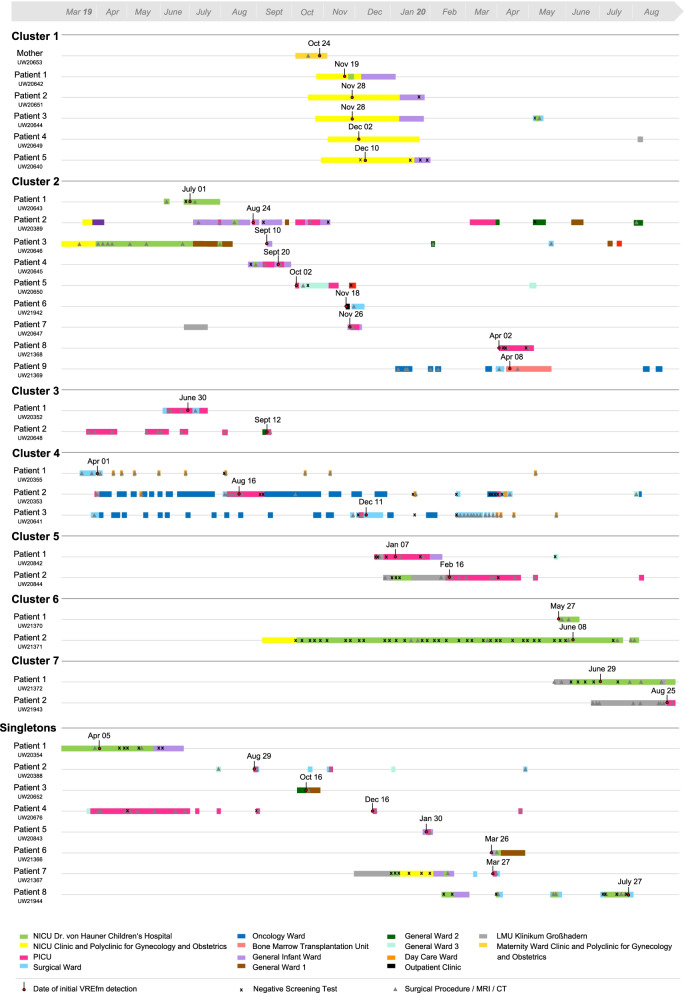
Timeline for each patient combining results from cgMLST with epidemiological and clinical data

Genome analysis (MLST) showed that VREfm isolates belonged to seven different STs. The most commonly detected STs were ST80 (n = 18), ST721 (n = 4), ST117 (n = 3), ST424 (n = 3) and ST1299 (n = 3). Regarding detection of resistance genes, PCR analysis identified *vanB* cluster in 54.5% (n = 18) and *vanA* cluster in 45.5% of isolates (n = 15). More detailed cgMLST analysis revealed genetic links, divided into seven distinct clusters (allele difference ≤ 15, Fig. 2). The clonal lineage ST80/CT1065 *vanB* represented the largest group, containing almost one third (n = 10) of all VREfm isolates. The combination of cgMLST with epidemiological and clinical data of the study population is shown in Fig. 3.

**Cluster 1** was found at the Polyclinic for Gynecology and Obstetrics. It contained five premature babies and a mother, who had been hospitalised due to complications with twin pregnancy. The mother (corresponding VREfm isolate ID UW20653, see Fig. 2 and 3), screened positive for VREfm on 24 October 2019, representing the initial case of this cluster. The first newborn (UW20642) was found to be colonised on 19 November, followed by both twins of the initial case (UW20651, UW20644) on 28 November. Subsequently, two additional patients (UW20649, UW20640) were screened positive beginning of December. All newborns were inpatients during the same time.

**Cluster 2** contained nine different isolates, detected within a period of ten months. Regarding the clinical history of affected patients, four children (patients 1, 4, 5, 8; UW20643, UW20645, UW20650, UW21368) had already been tested positive for VREfm during a previous external hospitalisation. After admission to Dr. von Hauner Children’s Hospital, patient 1 was an inpatient on NICU during the same time (June 2019) as patient 3 (UW20646), patient 2 (UW20389) was an inpatient on the general infant ward during the same time (August/September 2019) as patient 4 and patients 2 and 3 both had a gastroscopy on the same day (29 July 2019) performed by the same surgical team. Apart from two negative test results from patients 1 and 4, no screening tests were performed before initial VREfm detection.

**Cluster 3** occurred on PICU. The first colonised patient (UW20352) was a child with a malignant solid tumour, who was transferred to Munich for elective surgical treatment in June 2019. Seventy-four days after the primal case a second patient (UW20648) was screened positive for VREfm. In both cases, no screening tests were performed before initial detection of the respective bacterial strain.

Another three isolates, identified in 2019 within a period of nine months, built **Cluster 4**. All three affected children had an underlying oncological disease but were present on different wards when they were first screened VREfm positive. Except for one negative test result (patient 3; UW20641), no screening tests were performed before initial detection. Patients 1 (UW20355) and 3 were hospitalised at the same surgical ward end of March/beginning of April and patient 2 (UW20353) was admitted several times to the oncology ward for chemotherapy during the same period as patient 3. In addition, patients 1 and 2 had a surgical procedure one day apart (01 April and 31 March) performed by the same surgeon.

**Cluster 5** was detected on PICU in January/February 2020. Two patients (UW20842, UW20844) were screened positive for VREfm within 40 days. Before initial detection the patients were screened negative several times, however, patient 2 was temporarily hospitalised at another site of the LMU Klinikum, where no data for potential screening tests were available.

Further two isolates formed **Cluster 6**. The initial case (UW21370) in this cluster was an infant screened positive for VREfm on first day of admission (27 May 2020) to the NICU of Dr. von Hauner Children’s Hospital. A second patient (UW21371) was found to be colonised on 08 June, located at the same hospital ward during the same time as the initial case. Before detection of VREfm, patient 2 was screened negative several times.

**Cluster 7** included two isolates. The first patient (UW21372) with detected VREfm on 29 June 2020, was a child suffering from cardiac defect and severe postischemic brain injury who was transferred from another site of the LMU Klinikum to the NICU of Dr. von Hauner Children’s Hospital for further treatment. Before initial detection, the patient was tested negative several times. Patient 2 (UW21943) was screened positive on first day of admission to PICU on 25 August 2020. Regarding clinical history, patient 2 had evidence for VREfm colonisation during a previous hospital stay (10 August 2020) at another site of the LMU Klinikum, where patient 1 had been present two months before. In addition, patient 1 had a temporal overlap regarding hospital ward with both patients from **Cluster 6**.

Eight remaining isolates were considered **singletons** (a single clone that has no close relatives in the cgMLST). However, clinical-epidemiological data revealed evidence for a possible link between several cases (Fig. 2). The hospitalisation of patient 1 (UW20354) was congruent with the hospitalisation of patient 3 (UW20646) from **Cluster 2**. Patient 4 (UW20676) was present on PICU during the same time as both patients (UW20352, UW20648) from **Cluster 3** and patients 7 (UW21367) and 8 (UW21944) showed an epidemiological connection in terms of hospital wards with children (UW20844, UW21370, UW21371, UW21372) from **Cluster 5–7**. A temporal and personnel concordance in several performed surgical procedures presented another potential connection for some patients with no cluster assignment.

## Discussion

In this retrospective study, we aimed to investigate the accumulation of VREfm isolates during April 2019 and August 2020 at Dr. von Hauner Children’s Hospital. The main objective was to investigate a clonal spread, determine possible nosocomial transmission routes and analyse the affected population while in parallel evaluating previously described host risk factors for VREfm carriage or infection. In total, we found 33 children to be VREfm positive during the study period. CgMLST of all isolates revealed a polyclonal structure with a suspected spread demonstrated within seven distinct clusters and eight singletons. In combination with epidemiological and clinical data, our observations provided compelling evidence for transmission of VREfm between patients within the hospital.

**Cluster 1, 3, 5**, **6** and **7** consisted of isolates from children, who were present on identical hospital wards either during the same time or during a period following shortly thereafter (maximum seven weeks). These findings suggest a likely nosocomial transmission—a frequent and relevant issue that has been described in particular for VREfm compared to other multidrug-resistant microorganisms [41]. This fits well with the current state of research, where direct transmission between colonised or infected patients as well as indirect transmission via the patient’s surroundings are discussed as the most probable routes of spread [2, 10, 11, 42, 43]. Regarding the duration of VREfm-C in paediatric patients, periods ranging from several weeks to over six months are described [44–47]. Due to their extensive resilience, enterococci are known to survive even longer (up to several years) in hospital environments [1, 2]. Drees et al*.* found that patients admitted to rooms previously occupied by VRE carriers had a significantly higher risk of VRE acquisition [48]. Contaminated drip stands, ventilation tubes, incubators, thermometers, and other VRE positive surfaces were confirmed to play an important role in transmission pathways and multiple cleaning practices were shown to be inefficient for their decontamination [49–53]. In addition to transmission dynamics via the environment, these findings could be the result of close and constant interaction between patients and healthcare staff. Especially hands or gloves have been shown to act as a potential reservoir and transmission vehicle for nosocomial bacteria [9, 54, 55]. Moreover, we have shown that **Cluster 4** included VREfm positive patients, detected at different hospital wards. However, all three children had an underlying haemato-oncological disease and two patients had undergone a surgical procedure with the same surgical team one day apart from each other. These results indicate potential transmission via nursing staff or attending physicians in oncology or contamination of the hospital environment in general. **Cluster 2**, containing isolates from nine patients, showed a poor clinical-epidemiological linkage. Multiple children had been tested positive for VREfm during a previous external hospitalisation or had a positive test result on their first day of admission, assuming that they had already been colonised before initial detection at Dr. von Hauner Children’s Hospital. Nevertheless, we found some temporal overlap in terms of hospital wards or performed surgical procedures, again suggesting nosocomial transmission of VREfm.

Overall, our findings are in line with other reports, confirming VREfm transmission within and between wards by WGS/cgMLST and epidemiological data [10, 33, 56–58]. An inter-hospital spread can be assumed since the predominant clonal lineage in Bavaria ST80/CT1065 *vanB* represented the most commonly detected group (n = 10) at Dr. von Hauner Children’s Hospital [59]. Especially isolates of **Cluster 2** harboured the ST80/CT1065 *vanB* group, which may explain the poor clinical-epidemiological linkage and thus indicate a cross-contamination event with external hospitals. Frequent patient referrals between hospitals and specialities, in particular within different sites of the LMU Klinikum, may demonstrate a reason for the dissemination.

Interestingly, some of the genetically unrelated VREfm isolates showed a relevant connection in terms of epidemiological and clinical data of affected patients (patients of **Cluster 6** and **Cluster 7**; patients of the **singleton** group and **Cluster 2, 3, 5, 6** or **7**). In each case, VREfm isolates harboured *van*-genotypes, which were identical with genotypes of possible connected clusters (Fig. 2). This could refer to genetic mobility of *vanA* and *vanB* variants, allowing resistance to spread among different clonal lineages by horizontal gene transfer (HGT) [8, 60, 61]. Arredondo-Alonso et al. as well as Pinholt et al. previously identified high frequencies in HGT, especially of the *vanA* transposon Tn*1546* and corresponding *vanA* plasmids among unrelated *E. faecium* isolates as an alternative route of vancomycin resistance transmission in hospitals [62, 63].

To improve prevailing measures for the prevention of nosocomial VREfm spread at Dr. von Hauner Children’s Hospital, we evaluated affected patients, especially those with a presumed clinical infection requiring antibiotic therapy. Thereby, we have shown that more than half of all VREfm positive children were premature babies with young gestational age and low gestational weight. Regarding possible differences between VREfm-colonised and VREfm-infected patients, the presence of a temporary artificial stoma, a recent surgical procedure, previous treatment with carbapenems, preceding chemotherapy and underlying haemato-oncological disease were significantly associated with the development of clinical symptoms. These findings are in line with current studies, identifying preterm babies as well as immunosuppressed paediatric intensive care patients—in particular children with a haematological/oncological diagnosis—as a high-risk population for VREfm-C and VREfm-I [19, 20, 35, 64–67]. High exposure to antibiotics, especially third-generation cephalosporins, seems to further increase the risk [19, 35, 68].

Some important limitations need to be taken into account when interpreting our results. First, our study lacked negative test results in some patients prior to initial VREfm detection making it difficult to determine the exact time point of VREfm acquisition. This temporal imprecision may have influenced the accuracy of epidemiological data. Furthermore, we neither had information on patients’ room numbers or bays on NICU/PICU (to identify direct roommates) nor were samples of the patients’ environment available. Therefore, we were only able to make statements on ward level. Transmission via environmental contamination or healthcare staff (hands/gloves) could only be assumed, not confirmed. Second, it must be noted that clonal lineages such as ST80/CT1065 seem to have a very stable core genome (low allele differences), which often leads to the formation of clusters in cgMLST with no epidemiological link (neither temporally nor spatially) [59]. Third, focusing on cgMLST may have led to miss the confirmation of potential epidemiological links as a result of an overestimation of non-related isolates by excluding the analysis of possible HGT [62]. A polyclonal VREfm colonisation (also as a result of HGT) is conceivable, as usually only one colony (1 clone) per patient and time point is microbiologically processed, which does not necessarily reflect the totality of possible colonisation.

However, our findings regarding VREfm spread are still relevant and valuable. Future efforts should and will aim on exploring new and better ways to reduce nosocomial transmission events at Dr. von Hauner Children’s Hospital. In essence, the majority of existing international recommendations on the prevention of VREfm-C and VREfm-I in hospital call for improved hygiene measures, educational activities and screening as key interventions in suspected outbreak scenarios in clinical settings [69–74]. Following this study’s outbreak investigation it is essential to continuously review adherence to basic (hand) hygiene measures, in particular to the implementation of the “Five Moments for Hand Hygiene” [69–73]. Furthermore, we will aim to establish a new action plan consisting of a prevention bundle tailored to our affected population. Considering our findings we conclude that the bundle should include an active screening of rectal swabs for high-risk patients namely children and infants on intensive care units, surgical wards, the oncology ward and the bone marrow transplantation unit as well as a passive screening of every specimen taken for clinical indication [69–72, 74]. Routine surveillance of women with a high-risk pregnancy and high prenatal antibiotic use hospitalised at the Clinic and Polyclinic for Gynecology and Obstetrics should be considered. To achieve a reduction in clonal spread and horizontal transmission events, screening should be performed at regular intervals beginning on the first day of hospital admission [74, 75]. A subsequent isolation strategy for every VRE carrier including individual sanitary facilities for older children and mothers as well as enhanced barrier measures (gowns/gloves) for all contact persons constitute meaningful measures to reduce nosocomial VREfm dissemination [69–72, 75–77]. Improved cleaning and disinfection methods during and after hospitalisation of VREfm carriers and the involvement of affected patients and accompanying persons in hygiene measures can magnify the effect [50, 70–72, 74, 78]. Complementary to our current standard hygiene measures (disinfectant: Kohrsolin FF 0.5% or Terralin protect 0.5%) we consider to use ultraviolet-C (UV-C) light as an additional method to enhance terminal disinfection of patient rooms [79]. UV-C radiation has been used after detection of Cluster 1 at the Clinic and Polyclinic for Gynecology and Obstetrics. In general, it has been shown that efforts to reduce the use of unnecessary antibiotics are key to avoid selection of multidrug-resistant pathogens [80]. Despite no consistent scientific consensus about the impact of antibiotic stewardship programs (ASP) on VRE acquisition in general, an implementation in paediatric patients seems to be promising [69, 81, 82]. Further studies should regularly monitor the effectiveness of infection control measures and adherence to respective policies using defined suitable target variables. Finally, refinements to the examination of genomic data by a new approach that also includes the analyses of HGT mobilisation and polyclonal colonisation to effectively confirm potential epidemiological links may provide more accurate results for surveillance [62, 83].

## Conclusions

In conclusion, the Dr. von Hauner Children’s Hospital witnessed a substantial increase in the detection of VREfm isolates between April 2019 and August 2020, a dynamic that can be—at least in part—attributed to suggested nosocomial transmission events. Our study highlights the importance of protecting intensive care patients, who were mainly affected by the outbreak. In view of the monocentric character of this study, results may not entirely be generalisable to other clinical settings. However, findings and conclusions can serve as an example for comparable paediatric tertiary teaching hospitals. To achieve a reduction of transmission it is critical to further investigate VREfm genetic profiles and epidemiological links between colonised/infected patients, hospital environment and healthcare staff. Additional prospective studies are needed to continuously improve preventive efforts in hygiene measures, infection control and ASP to combat the spread of VREfm between hospitalised children and infants in Germany.

## Data Availability

The datasets generated and analysed during the current study are available from the corresponding author on reasonable request.

## Abbreviations

ASP: Antibiotic stewardship program
AWaRe: Access Watch Reserve
cgMLST: Core genome multilocus sequence typing
CT: Complex type
*E. faecalis*: *Enterococcus faecalis*
*E. faecium*: *Enterococcus faecium*
EUCAST: European Committee on Antimicrobial Susceptibility Testing
HGT: Horizontal gene transfer
LMU: Ludwig-Maximilians-University
MLST: Multilocus sequence typing
MST: Minimum Spanning Tree
NICU: Neonatal intensive care unit
PCR: Polymerase chain reaction
PICU: Paediatric intensive care unit
RKI: Robert Koch Institute
ST: Sequence type
UV-C: Ultraviolet-C
VRE: Vancomycin-resistant enterococci
VREfm: Vancomycin-resistant *Enterococcus faecium*
VREfm-C: VREfm colonisation
VREfm-I: VREfm infection
WGS: Whole-genome sequencing
WHO: World Health Organization
